# Crosstalk between PPARγ Ligands and Inflammatory-Related Pathways in Natural T-Regulatory Cells from Type 1 Diabetes Mouse Model

**DOI:** 10.3390/biom8040135

**Published:** 2018-11-05

**Authors:** S. Zulkafli Nor Effa, Nik Soriani Yaacob, Norazmi Mohd Nor

**Affiliations:** 1 School of Health Sciences, Universiti Sains Malaysia, Kelantan, Kubang Kerian 16150, Malaysia; 2 Regenerative Medicine Cluster, Advanced Medical and Dental Institute (AMDI), Universiti Sains Malaysia, Bertam, Kepala Batas 13200, Malaysia; 3 School of Medical Sciences, Universiti Sains Malaysia, Kelantan, Kubang Kerian 16150, Malaysia; niksoriani@usm.my

**Keywords:** T-Regulatory cells, immune regulation, Foxp3, PPARγ, autoimmune diabetes, NOD mouse, thiazolidinediones, ciglitazone, immunomodulator

## Abstract

Immunomodulation, as a means of immunotherapy, has been studied in major research and clinical laboratories for many years. T-Regulatory (Treg) cell therapy is one of the modulators used in immunotherapy approaches. Similarly, nuclear receptor peroxisome proliferator activated receptor gamma (PPARγ) has extensively been shown to play a role as an immuno-modulator during inflammation. Given their mutual roles in downregulating the immune response, current study examined the influence of PPARγ ligands i.e., thiazolidinedione (TZD) class of drugs on Forkhead Box P3 (Foxp3) expression and possible crosstalk between PPARγ and nTreg cells of Non-Obese Diabetes (NOD) and Non-Obese Diabetes Resistant (NOR) mice. Results showed that TZD drug, ciglitazone and natural ligand of PPARγ 15d-prostaglandin downregulated Foxp3 expression in activated nTreg cells from both NOD and NOR mice. Interestingly, addition of the PPARγ inhibitor, GW9662 further downregulated Foxp3 expression in these cells from both mice. We also found that PPARγ ligands negatively regulate Foxp3 expression in activated nTreg cells via PPARγ-independent mechanism(s). These results demonstrate that both natural and synthetic PPARγ ligands capable of suppressing Foxp3 expression in activated nTreg cells of NOD and NOR mice. This may suggest that the effect of PPARγ ligands in modulating Foxp3 expression in activated nTreg cells is different from their reported effects on effector T cells. Given the capability to suppress Foxp3 gene, it is possible to be tested as immunomodulators in cancer-related studies. The co-lateral use of PPARγ ligands in nTreg cells in inducing tolerance towards pseudo-self antigens as in tumor microenvironment may uphold beneficial outcomes.

## 1. Introduction

Over the past two decades since the identification of naturally-occurring CD4^+^CD25^+^Foxp3^+^ regulatory T cells (nTreg), there has been intense research in delineating the immunobiology of nTreg cells in physiological and pathological conditions [1]. The master regulator in nTreg cells is the transcription factor, Forkhead box P3 (Foxp3), which plays an important role in the development and function of these cells [2,3]. Foxp3 is expressed in the thymus by nTreg cells [4,5] and is transiently expressed by peripheral CD4^+^CD25^-^ conventional T cells (iTreg) [5]. In pathological conditions such as in autoimmune disorders, the recognition of self-tissues by auto-reactive T cells leads to the destruction of host tissues or organs. The immunosuppressive role of nTreg cells prevents such destruction from occurring by establishing peripheral self-tolerance toward these auto-reactive T cells [5]. This will thus hinder the development of debilitating autoimmune diseases from occurring. Hence mutation of Foxp3 gene results in the loss of immunoregulatory function of nTreg cells, predisposing the hosts towards autoimmune responses [3,4].

It is well established that Peroxisome Proliferator-Activated Receptor γ (PPARγ) activation is capable of inducing immunodownregulatory responses [6,7,8,9]. The ability of PPARγ in inducing anti-inflammatory responses in immune cells has been put forth. The activation of PPARγ by their ligands has been shown to downregulate the clonal expansion of activated T effector (Teff) cells [10]. PPARγ ligands such as the thiazolidinediones (TZDs) class of drugs alleviated adverse autoimmune conditions in allergic reactions and inflammatory bowel disease (IBD) [11,12]. In addition, PPARγ ligands have been shown to ameliorate multiple sclerosis [13,14]. The anti-inflammatory effect of 15d-prostaglandin-J2 (15d-PGJ2), a potent PPARγ ligand, significantly inhibited the activation of enchephalitogenic T cells and protected the development of encephalomyelitis in experimental autoimmune encephalomyelitis (EAE) [14]. The immunoregulatory function of PPARγ ligands may however act via PPARγ-dependent or -independent mechanisms [15]. The PPARγ ligand, pioglitazone, reduces allergic rhinitis in mice via PPARγ-dependent mechanism [16], while 15d-PGJ2 induces apoptosis in both T lymphocytes and Jurkat T cells via the activation of mitochondrial apoptotic pathway [17]. The correlation between PPARγ polymorphism with the increased risk of asthma in humans indicates that the role of PPARγ in anti-inflammatory reaction occurs at the gene expression level [18]. However, the possible mechanism of immunomodulation by PPARγ in nTreg cells is poorly understood. Therefore, this study was conducted to examine the possible crosstalk between immunoregulatory properties of nTreg cells and PPARγ in the murine autoimmune model of Non-Obese Diabetes (NOD) mice. We also included normal control strain of the autoimmune mouse model, Non-Obese diabetic resistant (NOR) mice.

## 2. Experimental Section

### 2.1. Mice

Eight-week old female NOD and NOR mice were purchased from The Jackson Laboratory (Bar Harbour, ME, USA). The mice were maintained in the animal facilities under specific pathogen-free conditions in accordance with the guidelines and regulations of the Universiti Sains Malaysia and used at 12-week of age. Peripheral blood glucose levels were measured every week. Any NOD mice with peripheral glucose level of 12 mmol/L and above for two consecutive weeks were considered as diabetic NOD and will be sacrificed for the study. All experimental protocols were approved by the Universiti Sains Malaysia (USM) Animal Ethics Committee (approval number: USM/Animal Ethics Approval/2009/(43 [132]). 

### 2.2. Antibodies and Reagents

Mouse nTreg cells were isolated from spleen tissues of NOD and NOR mice by magnetic separation. Briefly, CD4^+^ cells were purified by negative and positive isolations using MACS CD4^+^CD25^+^ Treg isolation kit (Miltenyi Biotec, Cologne, Germany). Isolated CD4^+^CD25^+^ Treg cells were then stained with phycoerythrin (PE)-anti mouse CD4, fluorescein isothiocyanate (FITC)-anti mouse CD25 and allophycocyanin (APC)-anti Foxp3 mAbs to determine cell purity using the fluorescence-activated cell sorting (FACS) Canto flow cytometer (BD Biosciences, Franklin Lakes, NJ, USA). Fixation and permeabilization of cells were prepared from BD Pharmingen™ Transcription Factor Buffer Set. Fix/Perm 1X working solution was freshly prepared from 1 part of 4X Fix/Perm buffer mixed well with 3 parts of diluent buffer and kept in dark at 4 °C until used. Working solution of 1X Perm/Wash buffer was diluted 1 part of 5X Perm/Wash buffer with 4 parts of sterile deionized water and kept at 4 °C. Labelled cells comprised ≥90% with >80% Foxp3^+^CD4^+^CD25^+^ cells were obtained. The isolated CD4^+^CD25^+^Foxp3^+^ cells were used as nTreg cells and were cultured in RPMI 1640 supplemented with 10% FBS (Thermo Fischer Scientific, Waltham, MA, USA), 5 ng/mL rmIL-2, 10 mM HEPES, 500 μL antibiotic stock solution containing 100 U/mL, 100 μg/mL streptomycin and 10 μM β-mercaptoethanol (Gibco BRL, Grand Island, NY, USA). IL-2 was purchased from BD Biosciences (New Jersey, NJ, USA), ciglitazone and 15d-PGJ2 were purchased from Cayman Chemicals (Ann Arbor, MI, USA) and GW9662 was purchased from Sigma-Aldrich (St. Louis, MO, USA).

### 2.3. Flow Cytometry Analysis

The expression of CD4 and CD25 surface markers and intracellular Foxp3 was evaluated using PE-conjugated anti-CD4, FITC-conjugated anti-CD25 and APC-conjugated anti-Foxp3. The phosphorylated ZAP-70 and STAT-5 proteins were evaluated by using FITC-conjugated anti-phosphorylated ZAP-70 and FITC-conjugated anti-phosphorylated STAT-5 antibodies and was assessed on FACS Canto Flow cytometer (BD Biosciences, NJ, USA). Mouse PE-and APC-conjugated IgG1 and FITC-conjugated IgG2a were used as isotype controls. Doublet discrimination and annexin analyses were not performed in this experiment due to low cell numbers and proliferation analysis was done using Carboxyfluorescein succinimidyl ester (CFSE) dye staining to observe expansion capacity of cultured cells (data not shown).

### 2.4. Total RNA Isolation, cDNA Synthesis and Real-Time PCR for the Detection of PPARγ and Foxp3

Total RNA was extracted using the RNAeasy Mini kit (Qiagen, Germany) and subjected to cDNA synthesis using cDNA first strand synthesis (Qiagen, Germany). To quantify the concentration of unknown transcripts, a standard curve for PPARγ was generated. Briefly, a serial dilution of the target PPARγ DNA plasmid concentration, corresponding to 101 to 107 copy numbers was prepared and amplified to generate standard curves. Polymerase Chain Reaction (PCR) was performed using primers for PPARγ (forward: 5′-GCG GCT GAG AAA TCA CGT TC-3′, Reverse: 5′-TTA AAA ATG TCC TGA ATA TCA GTG GTT C-3′, Probe: 5′-GCT TCT TTC AAA TCT TGT CTG TCA CAC AGT-3′). *Foxp3* gene with accession number AF277992.1 was selected from the National Center for Biotechnology Information (NCBI) database and quantified using TaqMan^®^ Gene Expression assay for *Foxp3* gene (assay ID Mm00475162_m1). The copy numbers for unknown samples were determined by extrapolating the data from these standard curves. The PPARγ expression level was reported as the number of mRNA transcripts per μg of total RNA (transcript/μg). Data were analyzed using the ABI prism software (Applied Biosystem, Foster City, CA, USA).

### 2.5. PPARγ-PPRE Binding Activity

PPARγ activation was measured by its binding to the response element, Peroxisome-proliferator response elements (PPRE). This was measured by using ligand binding assay of PPARγ transcription factors (Cayman Chemical, Ann Arbor, MI, USA). The nuclear proteins of treated and untreated cells were extracted using the Nuclear Extraction kit (Cayman Chemical, Ann Arbor, MI, USA). A 96 well-plate, pre-coated with immobilized PPRE was used to detect the binding of activated PPARγ in the nuclear extract from samples. Using rabbit polyclonal primary antibody against PPARγ and goat anti-rabbit secondary antibody-conjugated with horseradish peroxidase (HRP), the plate was prepared for detection of colorimetric signal changes using enzyme-linked immunosorbent assay (ELISA) plate reader at 450 nm.

### 2.6. Signaling Pathways Modulation by PCR Array

This experiment was conducted using real-time PCR and the data obtained were analyzed via close system software PCR Array Data Analysis Software provided by SA Biosciences (Qiagen, Germany). This software extrapolates the data based on *C*t values to report the fold differences between treated groups and untreated group using the 2^−∆∆*C*t^ formula. Total RNA was extracted from nTreg cells of NOD and NOR mice treated or untreated with PPARγ ligands. RNA extraction was performed using RNAeasy Mini kit (Qiagen, Germany) and subjected to cDNA synthesis using cDNA first strand synthesis (Qiagen, Germany). The cDNA was then used to perform Qiagen Mouse Signal Transduction Pathway Finder RT2 Profiler PCR Array (catalog no. PAMM-014), (Qiagen, Germany). Two Arrays were performed for each mouse strain. Changes in cycle threshold (∆*C*t) for each gene were obtained by subtracting the mean threshold cycle (*C*t) of the housekeeping genes (Gusb, HPRT, Hsp90ab1, Gapdh and β-Actin) from the threshold cycle value of the gene. The fold regulation was calculated relative to the untreated group when genes that showed more than 5-fold difference were determined as have been upregulated while those less than 0.2-fold considered as downregulated.

### 2.7. Statistical Analyses

Data from experimental analyses were presented as the mean of triplicates with standard error mean (mean ± SEM). The data were statistically analyzed using the Minitab^®^ 16.1.0 software (MiniTab Inc., State College, PA, USA). The comparison between control and treated groups was tested for significance using one-way analysis of variance (ANOVA) test. Post-Hoc comparison test was performed to compare significant levels between treated groups. *p* value of less than 0.05 (*p* < 0.05) is considered significant.

## 3. Results

### 3.1. Efficiency of CD4^+^CD25^+^Foxp3^+^ nTreg Cells Isolation from NOD and NOR Mice

**Figure 1:**
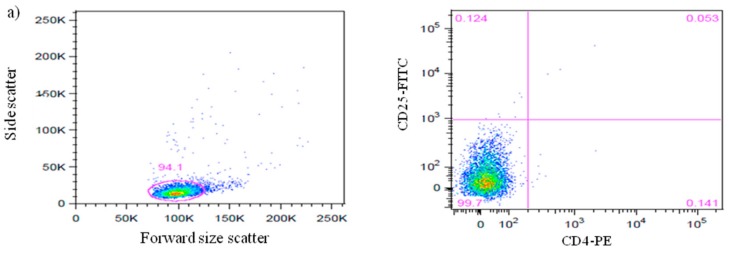

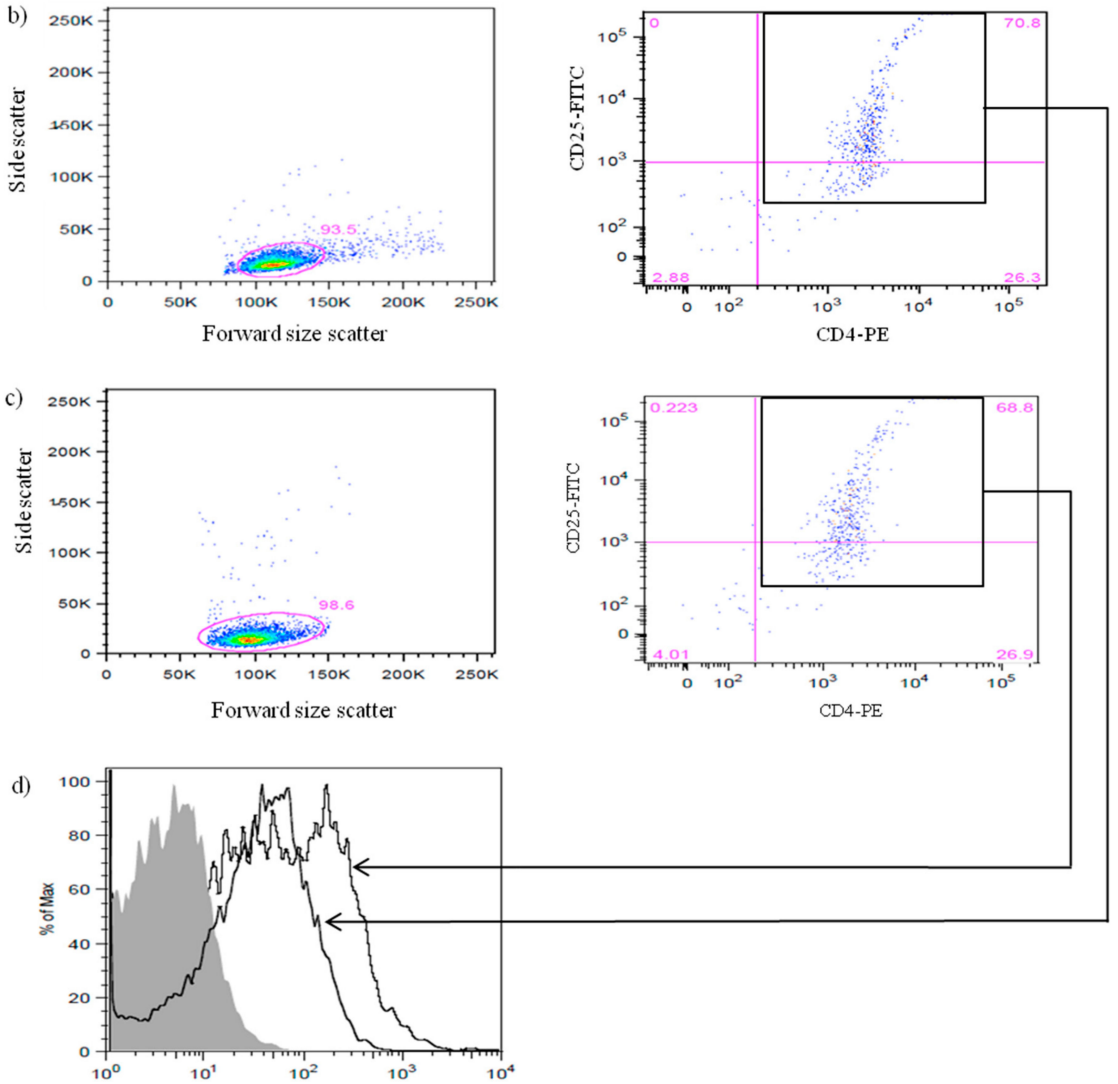
Efficiency of CD4^+^CD25^+^ expressing Foxp3+ Treg isolation from Non-Obese Diabetes (NOD) and Non-Obese Diabetes Resistant (NOR) mice splenocytes. (**a**) Dot plot representing lymphocytes stained with IgG1-PE and IgG2a-FITC isotype controls. (**b**) Dot plot shows lymphocytes stained with PE-conjugated rat anti-mouse CD4 and FITC-conjugated rat anti-mouse CD25 from NOD mice. (**c**) Dot plot shows lymphocytes stained with PE-conjugated rat anti mouse CD4 and FITC-conjugated rat anti-mouse CD25 from NOR mice. (**d**) Histograms show the expression of Foxp3^+^ cells gated on CD4^+^CD25^+^ populations from NOR mice (arrow) and NOD mice (arrow), compared with the isotype control (filled grey). Data shown are representative of three independent experiments. (*n* = 3 mice/experiment).

CD4^+^ cells with CD25^high^ and CD25^intermediate^ were considered as CD4^+^CD25^+^ cells. Enriched CD4^+^CD25^+^ homogenous cells were isolated for downstream experiments. The purity of nTreg cells isolated from splenocytes of NOD and NOR mouse strains was measured by flow cytometry (Figure 1). The percentage of CD4^+^CD25^+^ cells from both strains was >90% and was further stained with anti-Foxp3 antibodies to measure CD4^+^CD25^+^ cells that constitutively expressed Foxp3 protein. Figure 1d showed histogram of these cells expressing high intensity of Foxp3 protein with fluorescent intensity detected between 10^2^ to 10^3^ as compared to isotype control.

### 3.2. Expression of Foxp3 in nTreg Cells of NOD and NOR Mice

**Figure 2:**
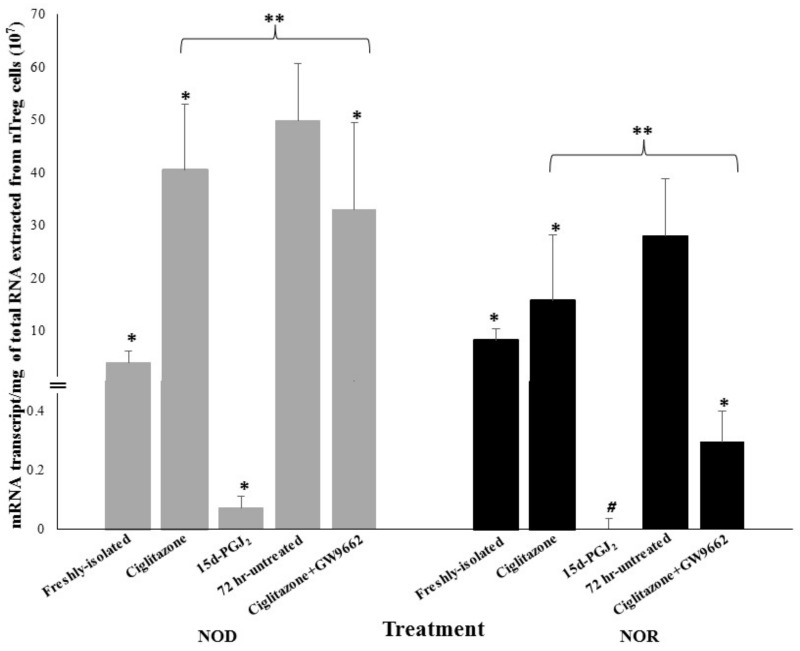
Foxp3 mRNA expression levels in nTreg cells from NOD and NOR mice following various treatment after 72 h culture. The grey bars represent Foxp3 mRNA transcripts from NOD mice while the black bars represent NOR mice. In NOD and NOR mice, the presence of ciglitazone significantly suppressed Foxp3 expression level in activated nTreg cells compared to untreated group. Moreover, the addition of GW9662 further downregulated Foxp3 level in these cells from both strains compared to untreated cells (*p* < 0.01) and ciglitazone-treated cells (*p* < 0.05). In NOD mice, Foxp3 expression in 15d-PGJ_2_-treated activated nTreg cells was suppressed compared to untreated cells (*p* < 0.01). Data are expressed as the amount of mRNA transcripts per μg of total RNA. This experiment was repeated twice and the graph was plotted based on the mean transcript values ± SEM. Statistical analysis was performed using One-way ANOVA. Post-hoc comparison was performed to identify the significance between treated samples (*n* = 4 mice/experiment). * *p* < 0.01, sample groups vs. untreated group. ** *p* < 0.05, ciglitazone-treated group vs. ciglitazone + GW9662-treated group. * *p* < 0.01, sample groups vs. untreated group. ** *p* < 0.05, ciglitazone-treated group vs. ciglitazone + GW9662-treated group. # the expression level was undetectable.

The influence of selected PPARγ ligands on Foxp3 in activated nTreg cells was tested by measuring the levels of Foxp3 mRNA expression in nTreg cells of NOD and NOR mice. These cells were treated with 15d-PGJ2 and ciglitazone in the presence or absence of the PPARγ inhibitor, GW9662. A group of untreated activated nTreg cells from both NOD and NOR mice was used as control for each of the mouse strain. The results obtained from the correlation analyses in NOD and NOR mice are shown in Figure 2. In both mouse strains, activated nTreg cells treated with ciglitazone expressed lower levels of Foxp3 mRNA compared to untreated group (*p* < 0.01). In both strains, the addition of GW9662, further reduced Foxp3 expression in these cells compared to ciglitazone-treated cells (*p* < 0.05). In NOD mice, treatment with 15d-PGJ2 significantly reduced the expression of Foxp3 mRNA in activated nTreg cells in comparison to untreated cells (*p* < 0.01). Similarly, Foxp3 expression in activated nTreg cells of NOR mice was undetectable after treatment with 15d-PGJ2 in comparison to untreated cells (*p* < 0.01).

### 3.3. Binding Activity between PPARγ and PPRE in nTreg Cells of NOD and NOR Mice

**Figure 3:**
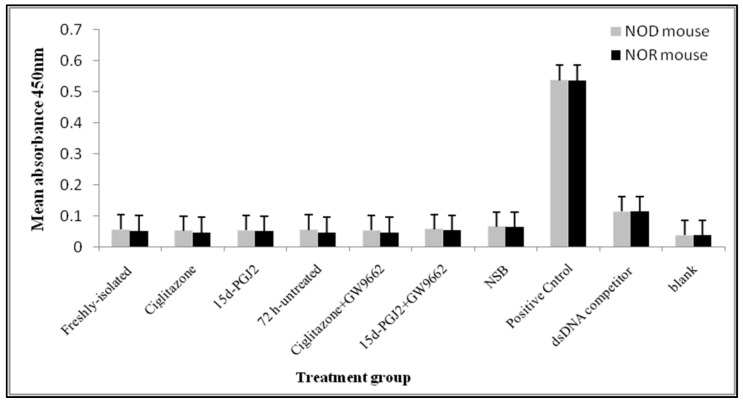
The binding activity between PPARγ and PPRE in nTreg cells nuclear protein lysates from NOD and NOR mice. The bar graphs show mean absorbance for each indicated treatment performed in duplicate. PPARγ double-stranded DNA (dsDNA) competitors were added onto dsDNA competitor wells while non-specific binding (NSB) wells were added with buffer without positive control or samples. Blank wells contained only buffer. This experiment was repeated twice and error bars represent mean ± SEM (*n* = 3 mice/experiment).

The specific sequence for PPARγ at the binding domain of DNA is known as PPRE. Activation of PPARγ via its ligand results in its translocation into the nucleus and the binding of the activated receptor to PPRE, at the DNA binding domain after heterodimerization with retinoid X receptor (RXR) [19]. Thus, we investigated the binding activity of PPARγ and PPRE in activated nTreg cells from NOD and NOR mice following treatment with their ligands, with or without the PPARγ inhibitor. For this analysis, we postulated that following treatment with PPARγ ligands i.e., ciglitazone and 15d-PGJ2, activated PPARγ would bind to PPRE. Our result showed that there was negligible binding activity between PPARγ and PPRE in activated nTreg cells from both NOD and NOR mice after the respective treatments (Figure 3). This suggests that the activity of PPARγ ligand treatment of nTreg cells is independent of PPRE binding. On the other hand, adipose tissue nuclear lysate which was used as a positive control showed strong binding with the plate-bound PPRE.

### 3.4. Phosphorylation Levels of ZAP-70 & STAT-5 Transduction Proteins

**Figure 4:**
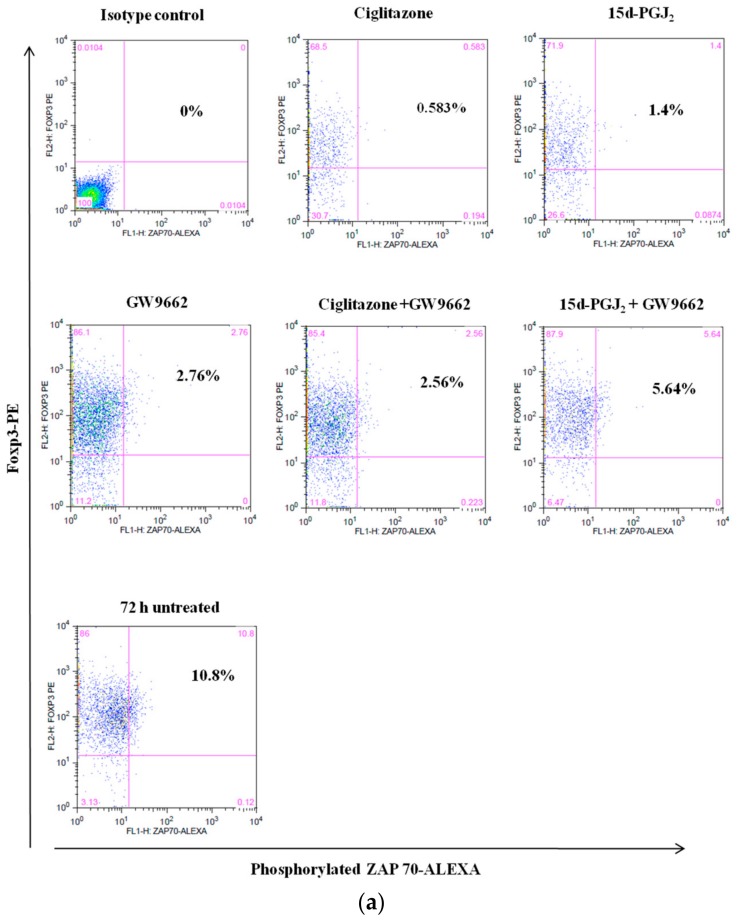

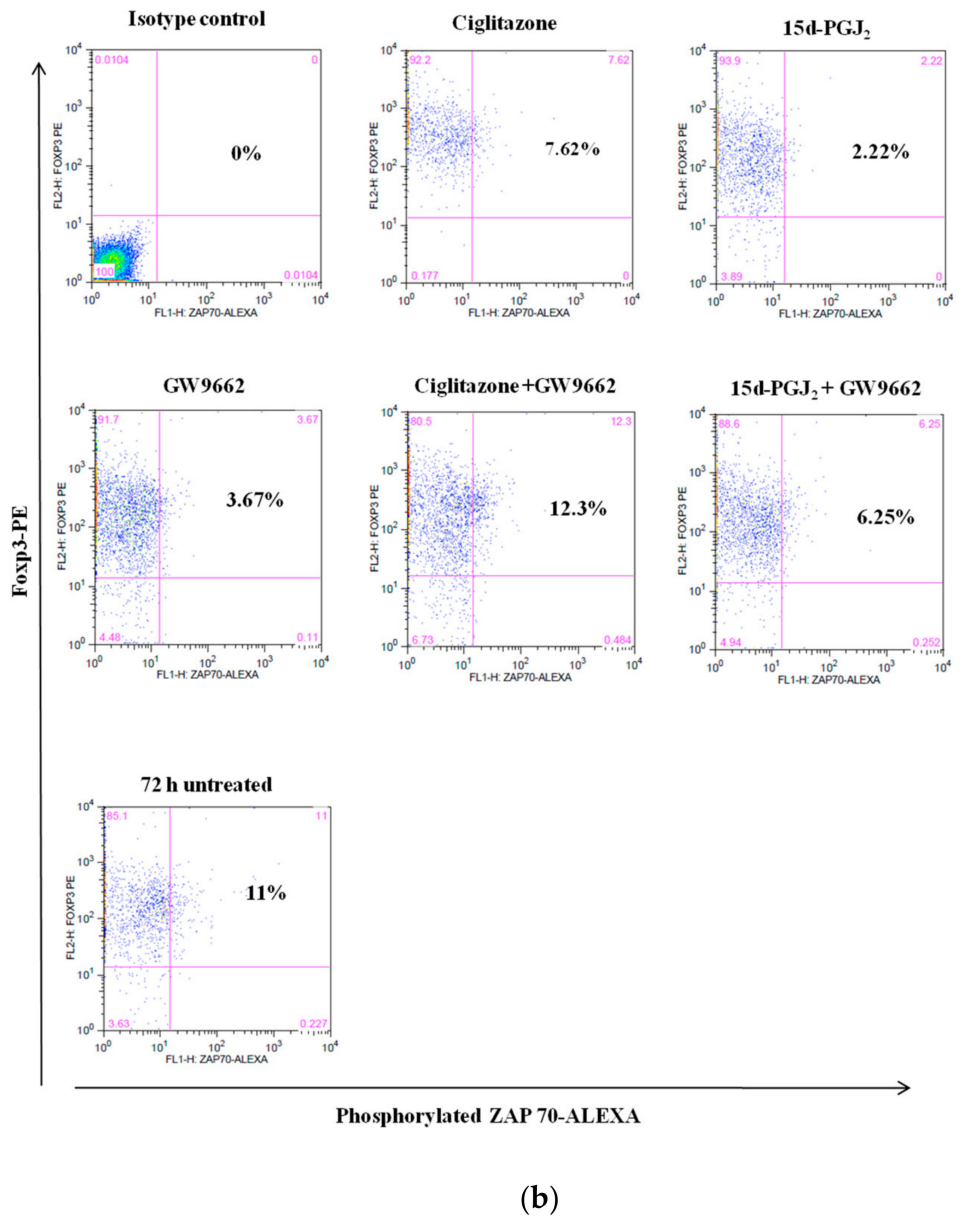
The expression of phosphorylated ZAP-70 in nTreg cells of (**a**) NOD and (**b**) NOR mice following various treatment after 72 h culture. Dot plot shows the levels of phosphorylated ZAP-70 in activated nTreg cells as indicated by Foxp3 expression. ZAP-70 phosphorylation was measured in nTreg cells treated with ciglitazone, 15d-PGJ_2_, GW9662, combination of ciglitazone + GW9662, and combination of 15d-PGJ_2_ + GW9662. 72 h-untreated cells were assigned as control group. Data shown are representative of two independent experiments. (*n* = 2 mice/experiment).

In newly activated T cells, phosphorylation of ZAP-70 initiates the activation of phosphorylation cascades in proximal intracellular signaling to propagate downstream responses. In order to examine the effect of PPARγ ligands on the proximal and distal signaling pathways in nTreg cells, phosphorylation levels of ZAP-70 proteins were studied in activated nTreg cells of NOD and NOR mice. Following 72 h incubation with the PPARγ ligands, nTreg cells were stained with phosphorylated-ZAP-70 conjugated antibodies. Untreated nTreg cells were included as controls and isotype control was used to set the marker for fluorochrome staining. There was no significant difference in the levels of ZAP-70 phosphorylation in either treated or untreated cells of NOD or NOR mice (Figure 4). These findings may suggest that ZAP-70 phosphorylation is not involved in the activation of nTreg cells of NOD and NOR mice.

**Figure 5:**
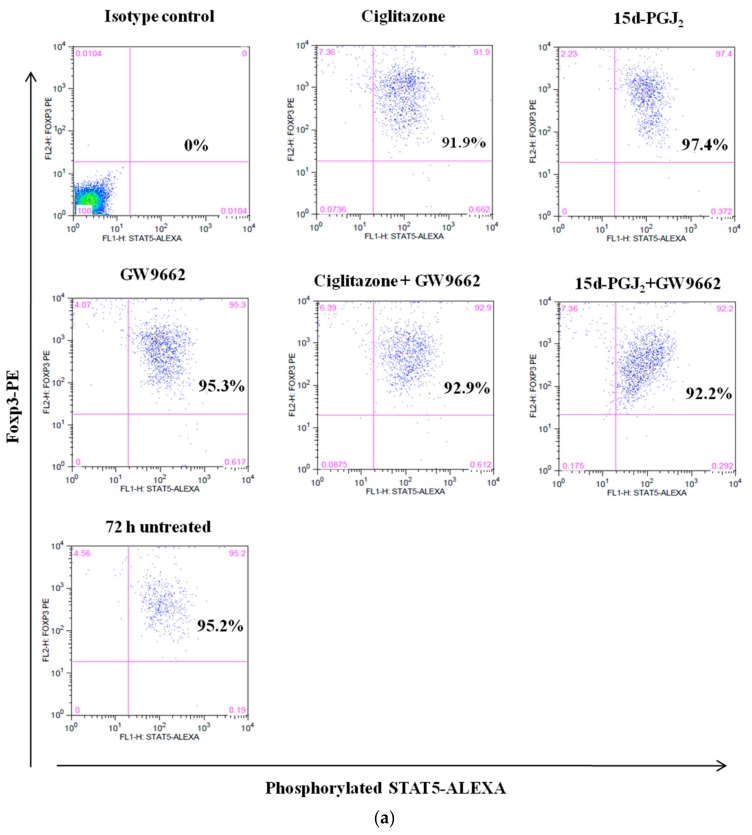

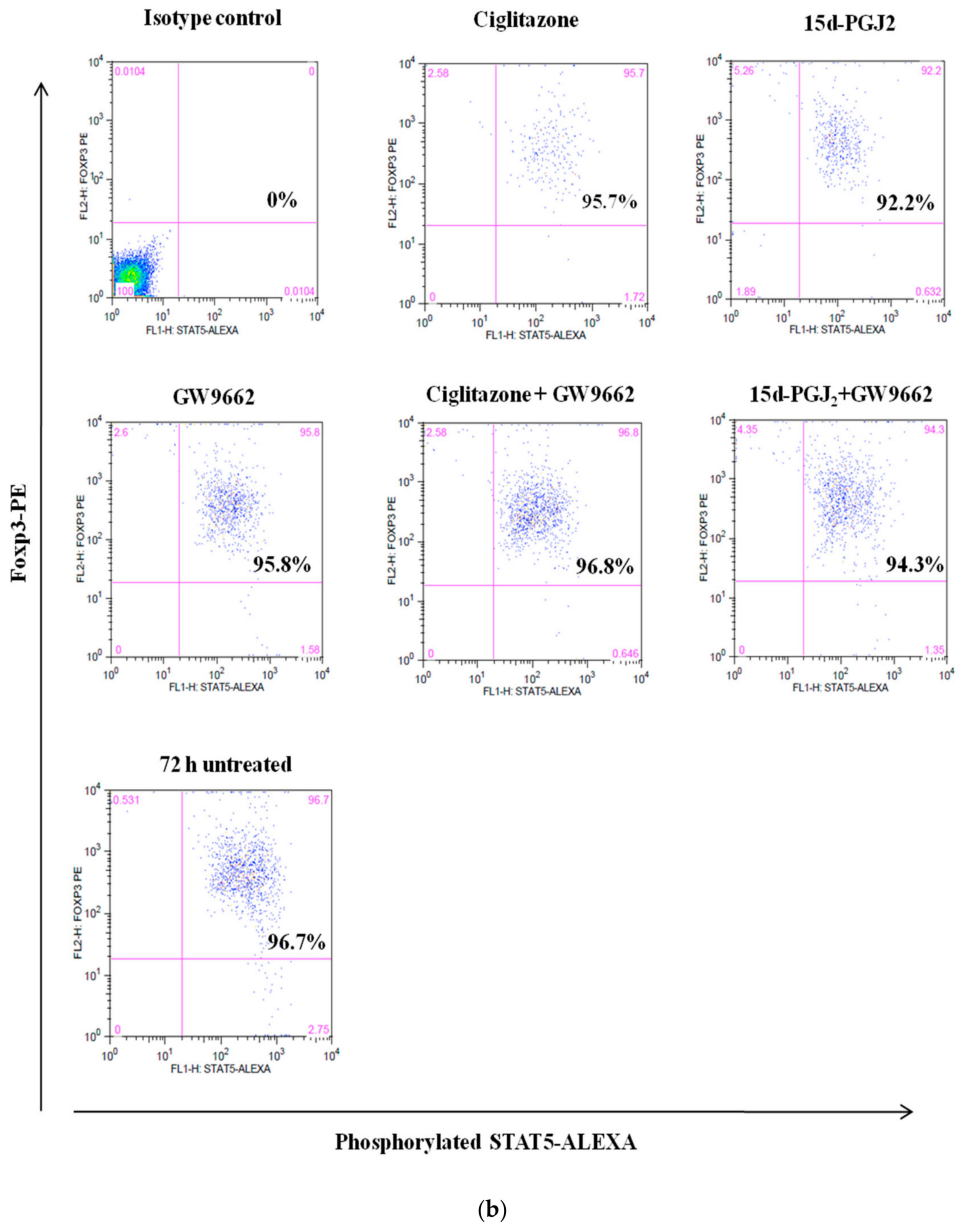
The expression of phosphorylated STAT-5 in activated nTreg cells of (**a**) NOD and (**b**) NOR mice following various treatment after 72 h culture. Dot plot shows the levels of phosphorylated STAT-5 in nTreg cells. STAT-5 phosphorylation level was measured in activated nTreg cells treated with ciglitazone, 15d-PGJ_2_, GW9662, combination of ciglitazone + GW9662, and combination of 15d-PGJ_2_ + GW9662. 72 h-untreated group was assigned as control group. Data shown are representative of two independent experiments (*n* = 2 mice/experiment).

In T-lymphocytes, IL-2 binding to IL-2R will activate STAT-5 transcription factor, which in turn activate a set of genes that are involved in T cell development and inflammation [20]. On the other hand, in nTreg cells, the activation of STAT-5 by IL-2 signaling is important in Foxp3 expression and survival of nTreg cells [21]. Upon IL2-IL2R binding, activation of STAT-5 by phosphorylation is initiated to affect downstream signaling components such as JAK/STAT signaling [22]. Therefore, IL-2/STAT-5 signaling pathway is an important pathway in nTreg cells as it directly correlates with Foxp3 expression. Intervention of this pathway by PPARγ ligands may indicate the association between PPARγ and nTreg cells at the IL-2/JAK-STAT level. Following 72 h incubation with the PPARγ ligands, these cells were stained with phosphorylated-STAT-5 conjugated antibodies. Similarly, untreated nTreg cells were included as controls and isotype control was used to set the marker for fluorochrome staining. Activated nTreg cells of NOD and NOR mice demonstrated constitutive phosphorylation of STAT-5 with no significant influence from PPARγ ligand treatment (Figure 5). Our current results may suggest that JAK/STAT signaling is required in activated nTreg cells for downstream events since we recorded high levels of phosphorylated STAT-5 in these cells. In addition, there was a negative association between STAT5 activation and PPARγ in nTreg cells of both NOD and NOR mice.

### 3.5. Differential Gene Expression Involved in Signal Transduction Pathways in nTreg Cells of NOD and NOR Mice 

In order to assess the possible crosstalk between PPARγ and signaling pathways in nTreg cells, we examined the modulation of selected gene expression in these cells following treatment with PPARγ ligands. By using the Signal Transduction PathwayFinder™ array, we analyzed 84 important genes responsive to 18 different signal transduction pathways. Out of the 18 signaling pathways, 4 signaling pathways that are relevant in immune cells were chosen.

The fold change cut-off value for gene expression was determined at >five-fold change in order to arbitrarily assign meaningful biological differences between expressed genes. This result is hoped to provide some information on biologically relevant genes in nTreg cells and their association with PPARγ. Tables S1–S4 depict the differentially regulated pathway-related target genes in NOD and NOR mice following treatment with PPARγ ligands. In NOD mice, most of inflammatory-related pathways were downregulated in PPARγ ligand-treated nTreg cells. In NOR mice, a few genes including *cdkn1a*, *ccl20*, *il2ra*, *lta*, *nfkbia*, *nos2* and *tank* genes were upregulated in PPARγ ligand-treated nTreg cells and these genes are known to be involved in pro- inflammatory related pathways. Furthermore, *cd5* gene that is responsible for the expression of CD5 antigen on cell surface was upregulated in ciglitazone-treated nTreg cells from NOR mice.

## 4. Discussion

Since both PPARγ and Foxp3 are involved in immune suppression, we investigated whether there is cross-talk between these transcription factors in nTreg cells. To provide insight into the functional aspect of these cells, we utilized the autoimmune diabetic, NOD mouse model, to further dissect the interactions between these transcription factors. PPARγ ligands have been shown to induce anti-inflammatory responses in autoimmune models [23,24,25]. However, whether PPARγ modulate the function of nTreg cells during autoimmune conditions has not been described.

Our analysis showed that PPARγ ligands downregulated Foxp3 expression in nTreg cells of both the autoimmune NOD as well as its control NOR mouse models. To further characterize whether this effect occurs via the PPARγ activation pathway, we treated the nTreg cells with its inhibitor, GW9662 and analyzed the binding of PPARγ to its response element. Our current data demonstrated that both ciglitazone and 15d-PGJ2 promoted the downregulation of Foxp3 expression independently of PPAR. The addition of GW9662 did not abrogate the effect of the PPARγ ligand, but instead further suppressed Foxp3 expression in nTreg cells of both NOD and NOR mice. This finding is in agreement with a previous report by Lei et al. [26] who showed that in human peripheral blood mononuclear cells, PPARγ agonists do not alter Foxp3 expression in nTreg cells and iTreg cells via PPRE. However, whether this effect is primarily due to the direct interaction between PPARγ and Foxp3 or secondary to interaction with other protein molecules require further investigation. The synergistic effect of PPARγ ligands and GW9662 have been demonstrated in growth inhibition of breast cancer cells [27] and suppression of IL-2 production in T-helper cells [28]. In fact, treatment with GW9662 alone was able to reduce IL-2 production in T-helper cells and Jurkat T-cell lines, suggesting that GW9662 has an intrinsic inhibitory property [28,29]. The reasons for these observations are however still unclear.

Our findings are contrary to previous reports that showed ciglitazone induced Foxp3 expression in in vitro generation of iTreg cells from PPARγ-deleted mouse model [30,31]. Since previous studies identified the role of these ligands on iTreg cells thus may contribute to discrepancy with current findings. In fact, previous study also reported that the suppressive capacity of nTreg cells is not affected by PPARγ ligands, an indication that PPARγ agonists neither modify the Foxp3 expression nor the suppressive function of nTreg cells in healthy animals [31]. This is in line with our data that showed Foxp3 protein levels in nTreg cells-treated with ciglitazone is not significantly suppressed as apposed mRNA levels that are significantly reduced following ligands treatment. The first report on the effect of PPARγ ligands on nTreg cells was demonstrated in a GHVD model [31] where co-transfer of wild type nTreg cells with ciglitazone positively promote survival of these mice compared to PPARγ -deleted nTreg cells. This suggests that PPARγ-expressing nTreg cell is important in mediating ciglitazone effect in vivo [31]. However, whether this effect is mediated by the level of Foxp3 or suppressive function in these cells was not studied. We speculate that the interaction between PPARγ and nTreg cells in in vivo setting may involve multiple players that results in the enhancement of nTreg cell function and that PPARγ ligands act differently in iTreg and nTreg cells respectively. Our study also demonstrated that, there was no significant PPARγ-PPRE interaction in PPARγ ligand-treated nTreg cells of both NOD and NOR mice, further indicating that the observed effect occurred independent of PPARγ activation.

PPARγ ligands negatively affect proinflammatory cytokine production by inhibiting the transcription factors AP-1, NF-κβ, NFAT and STATs in CD4^+^T cells [32,33,34]. On the other hand, Foxp3 expression requires binding of AP-1, NF-κβ and NFAT at the promoter and enhancer sites of nTreg cells [35,36,37,38]. Thus, the current findings suggest that PPARγ ligands may modulate Foxp3 expression in nTreg cells by interfering with the activation of these transcription factors which may in turn modulate the function of Foxp3 in nTreg cells. As a result, Foxp3 expression in nTreg cells is attenuated. We proposed that the effect is mediated by transrepression regulation of PPARγ ligands, which is differently described from the transcriptional suppression/repression mechanism [39,40].

We also examined whether this effect is due to PPARγ interference on the proximal or distal signaling components of nTreg cells by measuring the phosphorylation levels of ZAP-70 and STAT-5. The phospohorylation of ZAP-70 results in activation of downstream signaling pathways [41] while STAT-5 signaling is required for nTreg cell function and survival [21]. Therefore, we analyzed the level of phosphorylation of these mediators following treatment with PPARγ ligands. Our current study showed that PPARγ ligands did not modify the phosphorylation levels of ZAP-70 or STAT-5 in nTreg cells from both mice models. This finding suggests that these ligands do not suppress Foxp3 expression in nTreg cells from both mice models by modulating ZAP-70 and STAT-5 signaling pathways. These findings are consistent with a previous report by [42] where they elucidated that the activation of ZAP-70 is extremely low in CD4^+^CD25^+^Foxp3^+^ cells. Meanwhile, although not significantly different, STAT5 phosphorylation levels were reduced in nTreg cells from NOD mice following treatment with ciglitazone, and further reduced when PPARγ inhibitor were added. Conversely, 15d-PGJ_2_ induced STAT5 phosphorylation in nTreg cells from NOD mice and addition of PPARγ inhibitor reversed the effect. As opposed to NOD mice, nTreg cells showed the opposite effect of PPARγ ligands in NOR mouse model. Addition of both ligands reduced STAT5 phosphorylation and was slightly opposed by its inhibitor, GW9662. These findings may suggest the different regulation of PPARγ ligands in nTreg cells from NOD and NOR mice models. Previous study reported that PPARγ do not hinder STAT-5 phosphorylation but rather affect the downstream signaling of the growth hormone- activated STAT-5 activation [43].

Next, we investigated the differential effect of PPARγ ligands in these cells from NOD and NOR mice by examining any alterations of the relevant genes in signaling pathways using the PCR Array technology. While ciglitazone moderately induced myc and odc gene expression levels in nTreg cells of NOD and NOR mice, we also observed downregulation of pro- and anti-inflammatory pathway-related genes in PPARγ ligand-treated nTreg cells of NOD mice. The stimulation of myc gene indicates a common response to a vast group of substances that elicit phosphoinositate and activate PKC signaling pathway [44] whereas activation of odc1 gene promotes cell survival activities [45]. The activation of PKC pathway by ciglitazone is related on the survival of these cells in both mouse strains in vitro since PKC pathway activation in nTreg cells is canonical for cell proliferation and survival [46]. Furthermore, recent study showed that Foxp3 protein can regulate T cell metabolism by suppressing myc gene in T cell [47]. According to Angelin et al. (2017), Foxp3 suppresses myc-dependent genes and thus regulate Treg cell metabolism to adapt in low-glucose/high-lactate rich environments [47]. In the current findings, addition of ciglitazone inhibits Foxp3 expression in nTreg cells and induces myc expression in ciglitazone-treated nTreg cells in NOD and NOR mice. This may suggest that ciglitazone capable of reversing Foxp3 expression by upregulating myc expression in vitro.

Furthermore, we observed 12 target genes related to TGF-β, NF-κβ, and NFAT signaling pathways in PPARγ ligand-treated nTreg cells from NOR mice model. TGF-β and p53-related gene, *cdkn1a* that encodes protein cyclin-dependent kinase inhibitor was slightly upregulated in nTreg cells following treatment with ciglitazone, but not 15d-PGJ_2_. The upregulation may be due to cross-linkage with p53-dependent pathway since *cdkn1a* gene is under joint control of TGF-β and p53 signaling [48]. Also, overlapping cellular function by both components may indicate that they have potential convergence points in their signaling pathways [49]. Also, ciglitazone induced NF-κβ pathway-related genes i.e., *il2ra* and *nf-kbia*, but not 15d-PGJ_2_ in NOR mice. Other genes, such as *ccl20*, *lta* and tank were slightly upregulated in ciglitazone-treated cells. These data may suggest that ciglitazone restricts NF-κβ activation by upregulating *nf-kbia* expression, at the same time, inducing expression of CD25 molecules by upregulating *il-2ra* gene in nTreg cells from healthy NOR mice. The expression of this gene is also regulated by PKC signaling. Thus, upregulation of *il-2ra* gene induced by ciglitazone in nTreg cells is possibly via PKC dependent-NF-κβ pathway, since NF-β activation is preceded by PKC signaling. We also recorded that ciglitazone moderately upregulated NFAT-related target gene, *cd5* in nTreg cells from NOR mice. This gene is responsible to encode for CD5 molecules on nTreg cell surfaces, responsible for suppressive function [50] thus this may correspond to the default function of nTreg cells as immunosuppressor of autoreactive immune cells in normal condition.

In addition, we observed seven similar target genes that were downregulated in both NOD and NOR nTreg cells following treatment with PPARγ ligands. These target genes are *nab2* gene of MAPK pathway and *ei24* and *igfbp3* genes of NFAT pathway. MAPK-related target gene, *nab2* is associated with antigen recognition in CD4^+^ T lymphocytes [51]. Overall, the downregulation of pro- and anti-inflammatory related target genes by PPARγ ligands in NOD and NOR nTreg cells may suggest the correlation with Foxp3 expression in these cells in autoimmune diabetic NOD mice as well as healthy control NOR mice. Therefore, this data help in narrowing down possible regulatory molecules responsible on PPARγ-Foxp3 crosstalk in activated nTreg cells

Lastly, we would like to discuss on the possible technical variation in our laboratory settings. As this study isolated CD4^+^CD25^+^Foxp3^+^ cells using magnetic cell sorting method, thus we believe this could also contributed to the discrepancies in the findings. As some of the previous studies performed cell sorting by using automated method, this could also possible to differentially affect the cell phenotype and genotype when compared to magnetic method. Furthermore, since current study did not perform viability and doublet discrimination for flow cytometry analyses, we postulated that some of the cells are false positive lead to the impurity in cell isolation but at a manageable level as shown in the dot plot analyses.

## 5. Conclusions

Taken collectively, our current findings demonstrated the negative regulation of PPARγ ligands in inducing Foxp3 expression in nTreg cells of NOD and NOR mouse models. However, the underlying mechanism of Foxp3 suppression was differentially modulated in NOD and NOR nTreg cells. Our data from NOD and NOR mouse models suggest that PPARγ ligands attenuated Foxp3 mRNA expression in TCR-activated nTreg cells via PPARγ-independent pathways. Even though it is well-described that PPARγ ligands induce target gene expression via PPARγ-dependent manner, it is becoming increasingly clear that PPARγ ligands also mediate the non-genomic PPARγ-dependent and –independent manners [52,53]. The non-genomic PPARγ ligands requires activation of PPARγ by its ligands without subsequent DNA-binding of PPARγ [53]. This suggests that there are other possible mechanisms used by these ligands to regulate gene expression in ligand dependent but non PPRE-dependent manner by circumventing the binding to specific response elements in the promoter or enhancer region of target genes [39,40]. We speculate that in NOD nTreg cells, PPARγ ligands reduced Foxp3 expression either via downregulation of multiple target genes in pro-inflammatory pathway related genes. These pro-inflammatory signaling pathways such as NFAT, NK-κβ and PKC are essential for Foxp3 expression in nTreg cells, not for pro-inflammatory cytokine production [32,33,34,35,36,37,38]. An extensive analysis on Foxp3 expression in nTreg cells is also required in delineating the regulatory components involved in Foxp3 expression, particularly in committed nTreg cells. Although much remains to be learned about the basis of PPARγ and its ligands on Foxp3 expression in nTreg cells, the current study provided additional information to preliminary relationship between PPARγ ligands and Foxp3 expression thus put forward the redefinition of PPARγ ligands as immune modulators in tumor-associated conditions. In tumor microenvironment, population of Foxp3^+^Treg cells may indicate poor prognosis as they provide protection to tumor cells against tumor-infiltrating lymphocytes [54,55,56,57,58]. It is thus instrumental to look further into the potential function of TZD drugs on Foxp3^+^ Treg cells in tumor models as suggested by current study. The co-lateral use of PPARγ ligands in these cells in inducing tolerance towards pseudo-self antigens as in tumor microenvironment may uphold beneficial outcomes. 

## Supplementary Materials

The following are available online at http://www.mdpi.com/2218-273X/8/4/135/s1, Table S1: Differentially expressed target genes of pathways related to immune cells in ciglitazone-treated nTreg cells from NOD mice, Table S2: Differentially expressed target genes of pathways related to immune cells in prostaglandin J_2_-treated nTreg cells from NOD mice, Table S3: Differentially expressed target genes of pathways related to immune cells in ciglitazone-treated nTreg cells from NOR mice, Table S4: Differentially expressed target genes of pathways related to immune cells in prostaglandin J_2_-treated nTreg cells from NOR mice.
