# Supplementary material for: Crosstalk between PPARγ Ligands and Inflammatory-Related Pathways in Natural T-Regulatory Cells from Type 1 Diabetes Mouse Model
Source: Biomolecules. 2018 Nov 5;8(4):135. doi: 10.3390/biom8040135 (PMC6315476; doi:10.3390/biom8040135)
Supplement: Supplementary file 1 [file biomolecules-08-00135-s001.pdf]

biomolecules

Supplementary Material

Crosstalk between PPARγ Ligands and Inflammatory-Related Pathways in Natural T-Regulatory Cells from Type 1 Diabetes Mouse Model

S. Zulkafli Nor Effa [1,2,*], Nik Soriani Yaacob [3] and Mohd Nor Norazmi [1,*]

1 School of Health Sciences, Universiti Sains Malaysia, Kelantan, 16150 Kubang Kerian, Malaysia
2 Regenerative Medicine Cluster, Advanced Medical and Dental Institute (AMDI), Universiti Sains Malaysia, Bertam, 13200, Kepala Batas, Malaysia
3 School of Medical Sciences, Universiti Sains Malaysia, Kelantan, 16150 Kubang Kerian, Malaysia; niksoriani@usm.my
* Correspondence: effa@usm.my (S.Z.N.E.); norazmimn@usm.my (N.M.N.).

Table 1. Differentially expressed target genes of pathways related to immune cells in ciglitazone-treated nTreg cells from NOD mice.

No	Symbol	Description	GenBank Accession no	Unigene	Fold change[1]
**MAP KINASE**					
1	egr1	Early growth response 1	NM007913	Mm.181959	< 0.2
2	fos	FBJ osteosarcoma oncogene	NM010234	Mm.246513	< 0.2
3	jun	Jun oncogene	NM010591	Mm.275071	< 0.2
4	nab2	Ngfi-A binding protein 2	NM008668	Mm.336898	< 0.2
**TGF-β**					
5	cdkn1a	Cyclin dependent inhibitor 1A	NM007669	Mm.195663	< 0.2
6	cdkn1b	Cyclin dependent inhibitor 1B	NM009875	Mm.2958	< 0.2
7	cdkn2a	Cyclin dependent inhibitor 2A	NM0099877	Mm.4733	< 0.2
8	cdkn2b	Cyclin dependent inhibitor 2B, p15, inhibits CDK4	NM007670	Mm.423094	< 0.2
**NF-κβ**					
9	Ccl20	Chemokine (C-C) motif ligand 20	NM016960	Mm.116739	< 0.2
10	Cxcl1	Chemokine (C-X-C motif) ligand 1	NM008179	Mm.21013	< 0.2
11	Icam1	Intracellular adhesion molecule 1	NM010493	Mm.435508	< 0.2
12	Ikbkb	Inhibitor of kappa B kinase beta	Nm010546	Mm.277886	< 0.2
13	Il1a	Interleukin 1 alpha	NM010554	Mm.15534	< 0.2
14	Il2	Interleukin 2	Nm008366	Mm.14190	< 0.2
15	Il2ra	Interleukin 2 receptor alpha chain	NM008367	Mm.915	0.3686
16	Lta	Lymphotoxin A	NM010735	Mm.87787	< 0.2
17	Nfkbia	Nuclear factor of kappa light polypeptide gene enhancer in B-cells inhibitor, alpha	NM010907	Mm.170515	1.0718
18	Nos2	Nitric oxide synthase 2, inducible	NM010927	Mm.2893	< 0.2
19	Tank	TRAF-family-associated NF-k B activator	NM011529	Mm.244393	3.5540
20	Tnf	Tumor necrosis factor	NM013693	Mm.1293	0.3585
21	Vcam1	Vascular endothelial molecule 1	NM011693	Mm.76649	< 0.2
**NFAT**					
22	Cd5	CD5 antigen	NM007650	Mm.779	< 0.2
23	Fasl	Fas (TNF-receptor superfamily member 6) ligand	NM010177	Mm.3355	< 0.2
24	Il2	Interleukin 2	Nm008366	Mm.14190	< 0.2
**PKC**					
25	Csf2	Colony stimulating factor 2	NM009969	Mm.4922	< 0.2
26	myc	Myelocytomatosis oncogen	NM010849	Mm.2444	> 10.0
27	odc1	Ornithine decarboxylase, structural 1	NM013614	Mm.34102	> 5.0

[1] Fold change differences as compared to untreated control group. Fold change < 0.2 fold indicates downregulation and > 5 fold indicates upregulation. Genes with * are not differentially regulated as compared to control group.

Table 2. Differentially expressed target genes of pathways related to immune cells in prostaglandin J_2-treated nTreg cells from NOD mice.

No	Symbol	Description	GenBank Accession no	Unigene	Fold change[1]
**MAP KINASE**					
1	egr1	Early growth response 1	NM007913	Mm.181959	< 0.2
2	fos	FBJ osteosarcoma oncogene	NM010234	Mm.246513	< 0.2
3	jun	Jun oncogene	NM010591	Mm.275071	< 0.2
4	nab2	Ngfi-A binding protein 2	NM008668	Mm.336898	< 0.2
**TGF-β**					
5	cdkn1a	Cyclin dependent inhibitor 1A	NM007669	Mm.195663	< 0.2
6	cdkn1b	Cyclin dependent inhibitor 1B	NM009875	Mm.2958	0.60
7	cdkn2a	Cyclin dependent inhibitor 2A	NM0099877	Mm.4733	< 0.2
8	cdkn2b	Cyclin dependent inhibitor 2B, p15, inhibits CDK4	NM007670	Mm.423094	0.37
**NF-κβ**					
9	Ccl20	Chemokine (C-C) motif ligand 20	NM016960	Mm.116739	< 0.2
10	Cxcl1	Chemokine (C-X-C motif) ligand 1	NM008179	Mm.21013	< 0.2
11	Icam1	Intracellular adhesion molecule 1	NM010493	Mm.435508	< 0.2
12	Ikbkb	Inhibitor of kappa B kinase beta	Nm010546	Mm.277886	< 0.2
13	Il1a	Interleukin 1 alpha	NM010554	Mm.15534	< 0.2
14	Il2	Interleukin 2	Nm008366	Mm.14190	< 0.2
15	Il2ra	Interleukin 2 receptor alpha chain	NM008367	Mm.915	< 0.2
16	Lta	Lymphotoxin A	NM010735	Mm.87787	0.5620
17	Nfkbia	Nuclear factor of kappa light polypeptide gene enhancer in B-cells inhibitor, alpha	NM010907	Mm.170515	< 0.2
18	Nos2	Nitric oxide synthase 2, inducible	NM010927	Mm.2893	< 0.2
19	Tank	TRAF-family-associated NF-k B activator	NM011529	Mm.244393	< 0.2
20	Tnf	Tumor necrosis factor	NM013693	Mm.1293	1.2968
21	Vcam1	Vascular endothelial molecule 1	NM011693	Mm.76649	< 0.2
**NFAT**					
22	Cd5	CD5 antigen	NM007650	Mm.779	< 0.2
23	Fasl	Fas (TNF-receptor superfamily member 6) ligand	NM010177	Mm.3355	< 0.2
24	Il2	Interleukin 2	Nm008366	Mm.14190	< 0.2
**PKC**					
25	Csf2	Colony stimulating factor 2	NM009969	Mm.4922	*
26	myc	Myelocytomatosis oncogen	NM010849	Mm.2444	*
27	odc1	Ornithine decarboxylase, structural 1	NM013614	Mm.34102	> 5.0

[1] Fold change differences as compared to untreated control group. Fold change < 0.2 fold indicates downregulation and > 5 fold indicates upregulation. Genes with * are not differentially regulated as compared to control group.

Table 3. Differentially expressed target genes of pathways related to immune cells in ciglitazone-treated nTreg cells from NOR mice.

No	Symbol	Description	GenBank Accession no	Unigene	Fold change[1]
**MAP KINASE**					
1	egr1	Early growth response 1	NM007913	Mm.181959	3.650
2	fos	FBJ osteosarcoma oncogene	NM010234	Mm.246513	< 0.2
3	jun	Jun oncogene	NM010591	Mm.275071	< 0.2
4	nab2	Ngfi-A binding protein 2	NM008668	Mm.336898	< 0.2
**TGF-β**					
5	cdkn1a	Cyclin dependent inhibitor 1A	NM007669	Mm.195663	> 5.0
6	cdkn1b	Cyclin dependent inhibitor 1B	NM009875	Mm.2958	1.204
7	cdkn2a	Cyclin dependent inhibitor 2A	NM0099877	Mm.4733	< 0.2
8	cdkn2b	Cyclin dependent inhibitor 2B, p15, inhibits CDK4	NM007670	Mm.423094	< 0.2
**NF-κβ**					
9	Ccl20	Chemokine (C-C) motif ligand 20	NM016960	Mm.116739	> 5.0
10	Cxcl1	Chemokine (C-X-C motif) ligand 1	NM008179	Mm.21013	< 0.2
11	Icam1	Intracellular adhesion molecule 1	NM010493	Mm.435508	*
12	Ikbkb	Inhibitor of kappa B kinase beta	Nm010546	Mm.277886	*
13	Il1a	Interleukin 1 alpha	NM010554	Mm.15534	*
14	Il2	Interleukin 2	Nm008366	Mm.14190	*
15	Il2ra	Interleukin 2 receptor alpha chain	NM008367	Mm.915	> 5.0
16	Lta	Lymphotoxin A	NM010735	Mm.87787	> 5.0
17	Nfkbia	Nuclear factor of kappa light polypeptide gene enhancer in B-cells inhibitor, alpha	NM010907	Mm.170515	> 5.0
18	Nos2	Nitric oxide synthase 2, inducible	NM010927	Mm.2893	> 5.0
19	Tank	TRAF-family-associated NF-k B activator	NM011529	Mm.244393	> 5.0
20	Tnf	Tumor necrosis factor	NM013693	Mm.1293	*
21	Vcam1	Vascular endothelial molecule 1	NM011693	Mm.76649	*
**NFAT**					
22	Cd5	CD5 antigen	NM007650	Mm.779	> 5.0
23	Fasl	Fas (TNF-receptor superfamily member 6) ligand	NM010177	Mm.3355	*
24	Il2	Interleukin 2	Nm008366	Mm.14190	*
**PKC**					
25	Csf2	Colony stimulating factor 2	NM009969	Mm.4922	*
26	myc	Myelocytomatosis oncogen	NM010849	Mm.2444	> 5.0
27	odc1	Ornithine decarboxylase, structural 1	NM013614	Mm.34102	> 5.0

[1] Fold change differences as compared to untreated control group. Fold change < 0.2 fold indicates downregulation and > 5 fold indicates upregulation. Genes with * are not differentially regulated as compared to control group.

Table 4. Differentially expressed target genes of pathways related to immune cells in prostaglandin J_2-treated nTreg cells from NOR mice.

No	Symbol	Description	GenBank Accession no	Unigene	Fold change[1]
**MAP KINASE**					
1	egr1	Early growth response 1	NM007913	Mm.181959	1.501
2	fos	FBJ osteosarcoma oncogene	NM010234	Mm.246513	*
3	jun	Jun oncogene	NM010591	Mm.275071	2.020
4	nab2	Ngfi-A binding protein 2	NM008668	Mm.336898	< 0.2
**TGF-β**					
5	cdkn1a	Cyclin dependent inhibitor 1A	NM007669	Mm.195663	*
6	cdkn1b	Cyclin dependent inhibitor 1B	NM009875	Mm.2958	*
7	cdkn2a	Cyclin dependent inhibitor 2A	NM0099877	Mm.4733	*
8	cdkn2b	Cyclin dependent inhibitor 2B, p15, inhibits CDK4	NM007670	Mm.423094	*
**NF-κβ**					
9	Ccl20	Chemokine (C-C) motif ligand 20	NM016960	Mm.116739	0.4983
10	Cxcl1	Chemokine (C-X-C motif) ligand 1	NM008179	Mm.21013	0.1632
11	Icam1	Intracellular adhesion molecule 1	NM010493	Mm.435508	0.6713
12	Ikbkb	Inhibitor of kappa B kinase beta	Nm010546	Mm.277886	0.1632
13	Il1a	Interleukin 1 alpha	NM010554	Mm.15534	1.0317
14	Il2	Interleukin 2	Nm008366	Mm.14190	0.4846
15	Il2ra	Interleukin 2 receptor alpha chain	NM008367	Mm.915	0.695
16	Lta	Lymphotoxin A	NM010735	Mm.87787	0.3856
17	Nfkbia	Nuclear factor of kappa light polypeptide gene enhancer in B-cells inhibitor, alpha	NM010907	Mm.170515	2.6117
18	Nos2	Nitric oxide synthase 2, inducible	NM010927	Mm.2893	1.0755
19	Tank	TRAF-family-associated NF-kB activator	NM011529	Mm.244393	2.5758
20	Tnf	Tumor necrosis factor	NM013693	Mm.1293	1.0317
21	Vcam1	Vascular endothelial molecule 1	NM011693	Mm.76649	0.4132
**NFAT**					
22	Cd5	CD5 antigen	NM007650	Mm.779	2.2894
23	Fasl	Fas (TNF-receptor superfamily member 6) ligand	NM010177	Mm.3355	< 0.2
24	Il2	Interleukin 2	Nm008366	Mm.14190	< 0.2
**PKC**					
25	Csf2	Colony stimulating factor 2	NM009969	Mm.4922	*
26	myc	Myelocytomatosis oncogen	NM010849	Mm.2444	*
27	odc1	Ornithine decarboxylase, structural 1	NM013614	Mm.34102	> 5.0

[1] Fold change differences as compared to untreated control group. Fold change < 0.2 fold indicates downregulation and > 5 fold indicates upregulation. Genes with * are not differentially regulated as compared to control group.
